# The neuroanatomical correlates of anxiety in a healthy population: differences between the State-Trait Anxiety Inventory and the Hamilton Anxiety Rating Scale

**DOI:** 10.1002/brb3.232

**Published:** 2014-06-18

**Authors:** Giulia Donzuso, Antonio Cerasa, Maria C Gioia, Manuela Caracciolo, Aldo Quattrone

**Affiliations:** 1 IBFM, National Research CouncilCatanzaro, Italy; 2 Department “G.F. Ingrassia”, Section of Neuroscience, University of CataniaCatania, Italy; 3 Institute of Neurology, University “Magna Graecia”Germaneto, Italy

**Keywords:** Anterior cingulate cortex, cortical thickness, STAI and HARS scales, voxel-based morphometry

## Abstract

**Objectives:**

The State-Trait Anxiety Inventory (STAI) and the Hamilton scale for anxiety (HARS) are two of the most important scales employed in clinical and psychological realms for the evaluation of anxiety. Although the reliability and sensibility of these scales are widely demonstrated there is an open debate on what exactly their scores reflect. Neuroimaging provides the potential to validate the quality and reliability of clinical scales through the identification of specific biomarkers. For this reason, we evaluated the neural correlates of these two scales in a large cohort of healthy individuals using structural neuroimaging methods.

**Case report:**

Neuroimaging analysis included thickness/volume estimation of cortical and subcortical limbic structures, which were regressed on anxiety inventory scores with age and gender used for assessing discriminant validity. A total of 121 healthy subjects were evaluated. Despite the two anxiety scales, at a behavioral level, displaying significant correlations among them (HARS with STAI-state (*r* = 0.24; *P* = 0.006) and HARS with STAI-trait (*r* = 0.42; *P* < 0.001)), multivariate neuroimaging analyses demonstrated that anatomical variability in the anterior cingulate cortex was the best predictor of the HARS scores (all *β*'s ≥ 0.31 and *P*'s ≤ 0.01), whereas STAI-related measures did not show any significant relationship with regions of limbic circuits, but their scores were predicted by gender (all *β*'s ≥ 0.23 and *P*'s ≤ 0.02).

**Conclusion:**

Although the purpose of HARS and STAI is to quantify the degree and characteristics of anxiety-like behaviors, our neuroimaging data indicated that these scales are neurobiologically different, confirming that their scores might reflect different aspects of anxiety: the HARS is more related to subclinical expression of anxiety disorders, whereas the STAI captures sub-dimensions of personality linked to anxiety.

## Introduction

Anxiety may be defined as a group of emotional reactions caused by external or internal stimuli. Anxiety is a symptom that accompanies numerous psychological/psychiatric disorders, such as Generalized Anxiety Disorder (GAD) and Social Anxiety Disorder (SAD) (Torres et al. 2012; Uchida et al. 2008), and neurological disorders (i.e., Parkinson's disease, Mondolo et al. 2007).

Two of the most widely used measurements to assess anxiety are the State-Trait Anxiety Inventory (STAI) and Hamilton scale for anxiety (HARS) (Balon 2007; Spielberger 1989). The STAI is a self-administered assessment scale for the evaluation of severity of anxiety, that clearly differentiates between the temporary condition of state anxiety and the more general and long-standing quality of trait anxiety, each containing 20 items addressing somatic, affective, and cognitive aspects. According to Spielberger's anxiety theory (Spielberger et al. 1970) “state” is defined as a subjective, consciously perceived feeling of fear and tension that is accompanied by the activation or agitation of the autonomous nervous system. On the other hand, anxiety as a “trait” is defined as a motif or acquired behavioral disposition that makes an individual susceptible to perceiving a wide range of objectively harmless situations as threatening and to react to them with the anxiety states. Several lines of evidence have shown that STAI has an elevated construct validity since its scores are positively correlated with other scales that measure anxiety (HARS, Mondolo et al. 2007 and Anxiety Scale Questionnaire [ASQ]) and present high test–rest reliability (Rule and Traver 1983). Although several hundred published studies have employed this scale in nonclinical contexts, STAI is also widely used to clinically assess anxiety in psychiatric (Kennedy et al. 2001; Tecer et al. 2004) and neurological populations (Di Legge et al. 2003; Tinaz et al. 2011).

HARS is a clinician-administered scale to assess anxiety symptoms (Hamilton 1959), normally used in psychiatric contexts (Bech 2009). There are several versions of this clinician-rated scale, but the 14- and 15-item versions are the most used. Designed to measure the severity of anxiety, it contains a high proportion of somatic items.

STAI and HARS scales have a great sensitivity in detecting anxiety disorders and anxiety-like behaviors (Balon 2007; Bech 2011), although HARS is used to assess anxiety symptoms in a broad way (Bech 2011, 2012; de Bonis 1974), while STAI is usually used to capture enduring characteristics and patterns of symptoms (Campbell-Sills and Brown 2004). However, to date it is still unclear exactly what their scores reflect (Kennedy et al. 2001; Montag et al. 2013). The modern techniques of neuroimaging offer a way to consistently validate the quality and reliability of a behavioral/clinical scale through the identification of specific biomarkers. A biomarker is any measurable indicator (functional brain activity or morphological change) of a disease or behavior that could be correlated with a single aspect of the disease process. In other words, a biomarker is a characteristic that could be objectively measured and evaluated as an indicator of a normal biological process (Lenzenweger 2013). A large number of studies have explored the macro- and micro-structural biomarkers of anxiety-related processes. This research highlighted the presence of different degrees of relationship (positive and negative association) between the presence of anxiety in a nonclinical population as assessed by various clinical scales (STAI, Beck Anxiety Inventory (BAI), behavioral inhibition scale/behavioral approach system (BIS/BAS) and Liebowitz Social Anxiety Scale (LSAS)) and specific brain measurements. Overall, what clearly emerged from these studies was that the more important brain regions (biomarkers) involved in anxiety-like behaviors are: the amygdala, hippocampus, orbitofrontal cortex (OFC), and anterior cingulate cortex (ACC) (Blackmon et al. 2011; Baur et al. 2012; Kuhn et al. 2011; Spampinato et al. 2009; Liao et al. 2010; Fuentes et al. 2012; Barros-Loscertales et al. 2006; Cherbuin et al. 2008). As concerns HARS measurements, although this is normally employed in a clinical context (Zhang et al. 2013), some neuroimaging studies employed this scale for assessing anxiety in healthy populations (Rasetti et al. 2010; Schunck et al. 2008), without providing a definite neural correlates of this scale.

Individual differences in anxiety symptoms or traits may place certain people at greater risk for development of psychopathology or neuropsychiatric disorders. Therefore, studying the neural correlates may help us to understand neural mechanisms underlying risk behaviors in both clinical and nonclinical populations. Since neuroimaging literature about STAI and HARS measurements are somewhat confusing, this study is aimed at providing, on a large nonclinical population, the neuroanatomical correlates of these two important scales in order to identify risk factors for developing anxiety disorders throughout mechanisms linked with the morphology of critical regions involved in emotion regulation and control (amygdala, hippocampus, OFC, and ACC). To this end, we employed two well-known complementary structural neuroimaging metrics: voxel-based morphometry (VBM) (Ashburner and Friston 2005) and cortical thickness (Freesurfer) (Fischl and Dale 2000). The null hypothesis is that if these scales measure similar aspects of anxiety, neuroimaging data should provide similar neural correlates associated with their scores. Otherwise, the alternative hypothesis is whether HARS and STAI scores reflect different aspects of the biological mechanisms underlying anxiety-like behaviors; in this case their scores might be associated with different brain networks.

## Material and Methods

### Subjects

From April 2007 to June 2010, 159 right-handed healthy subjects were recruited from the University “Magna Græcia” of Catanzaro. All participants gave written informed consent for participation in the study, which was approved by the local ethics committee at the University of Catanzaro. Exclusion criteria were: (1) no evidence of neurological or psychiatric disorders (assessed with Structure Clinical Interview for Diagnostic and Statistical Manual of Mental Disorder-IV) (six individuals fulfilled this criteria); (2) no histories of substance abuse or other medical problems (three individuals fulfilled this criteria); (3) no presence of vascular brain lesions, brain tumor and/or marked cortical and/or subcortical atrophy on magnetic resonance imaging (MRI) scan (20 individuals fulfilled this criteria); and (4) no presence of cognitive impairment (Mini Mental State Examination (MMSE) score ≥ 24) (nine individuals fulfilled this criteria).

After a careful evaluation of these exclusion criteria, 121 subjects (mean ± SD age = 38.7 ± 15.1; 67 (55%) women) were eligible for this study. Eighty-three individuals had taken part in our previous imaging genetic studies (Labate et al. 2012; Cerasa et al. 2009; Liguori et al. 2007). Psychometric evaluation of anxiety was performed using the: (1) HARS test, a 14-item test measuring the severity of anxiety symptoms; each item is scored on a scale of 0 (not present) to 4 (severe), with a total score range of 0 to 56 with scores <17 suggesting mild anxiety, 18 to 24 mild to moderate anxiety and 25 to 30 moderate to severe anxiety, >30 severe anxiety; (b) STAI test (form Y), a 40-question self-administered test for measuring anxiety as a state (i.e., temporary and situational anxiety) or trait (i.e., general, long-standing proneness to anxious situations); the STAI scale has 20 questions each for state and trait anxiety, and each question is scored on a 4-point Likert-type scale, ranging from 1 to 4 (from “not at all” to “very much so” for the STAI-state and from “almost never” to “almost always” for the STAI-trait) with a total score range of 20 to 80, with scores >40 suggesting for pathological level of anxiety. Low scores indicate a mild form of anxiety whereas median scores indicate a moderate form of anxiety and high scores indicate a severe form of anxiety. The median alpha reliability coefficients for the State and Trait scales (Form Y) are 0.92 and 0.90, respectively (Spielberger et al. 1983). Similarly, HARS has been found to have an Inter-rater reliability, reported as an Intraclass Correlation Coefficient, of 0.74–0.96 (Bruss et al. 1994).

### MRI data acquisition

Brain Magnetic resonance imaging (MRI) was performed according to our routine protocol with a 1.5-T unit (Signa NV/I; GE Medical Systems, Milwaukee, WI). A 3D T1-weighted high-resolution spoiled gradient echo (SPGR) sequence with a 1.2-mm slice thickness and an isotropic in-plane resolution of 0.94 mm was acquired with the following parameters: repetition time 15.2 ms, echo time 6.7 ms, flip angle 15°, 115 slices, matrix size 256 × 256 and a field of view of 24 cm.

### Amygdala-hippocampus volumetry

Automated labeling and quantification of amygdala and hippocampal volumes were performed using FreeSurfer 4.05 installed on a Red Hat Enterprise Linux v.5. The automated procedures for volumetric measures of several deep GM structures have been previously described (Cerasa et al. 2011; Fischl et al. 2002). This procedure automatically provided segments and labels for up to 40 unique structures and assigned a neuroanatomical label to each voxel in an MRI volume based on probabilistic information estimated automatically from a manually labeled training set.

The automated subcortical segmentation performed by *Freesurfer* required the following steps. First, an optimal linear transform was computed that maximized the likelihood of the input image, given an atlas constructed from manually labeled images. A nonlinear transform was then initialized with the linear one, and the images were allowed to further deform to better match the atlas. Finally, a Bayesian segmentation procedure was performed, and the maximum a posteriori estimate of the labeling was computed. This approach provides advantages similar to manual region-of-interest (ROI) drawing (Jovicich et al. 2009; Morey et al. 2009) without the potential for rater bias, offering an anatomically accurate rendering of regional volumes. Intracranial volume (ICV) was calculated and used to correct the regional brain volume analyses.

### Cortical thickness analysis

Magnetic resonance imaging (MRI)-based quantification of cortical thickness was further performed using *Freesurfer*. This method has been previously described in detail (Fischl et al. 2002, 1999). The procedure involves segmentation of white matter, tessellation of the gray/white matter junction, inflation of the folded surface, tessellation patterns, and automatic correction of topological defects in the resulting mainfold. Cortical thickness measurements were obtained by reconstructing representations of the gray/white matter boundary and the cortical surface. The distance between these two surfaces was calculated individually at each point across the cortical mantle. This method uses both intensity and continuity information from the entire 3D MRI volume in segmentation and deformation procedures to construct representations of cortical thickness. Thickness measurements can be mapped onto the “inflated” surface of each participant's reconstructed brain, thus allowing visualization without interference from cortical folding. Maps were smoothed using a circularly symmetrical Gaussian kernel across the surface with a standard deviation of 10 mm and averaged across participants using a nonrigid high-dimensional spherical averaging method to align cortical folding patterns.

**Figure 1:**
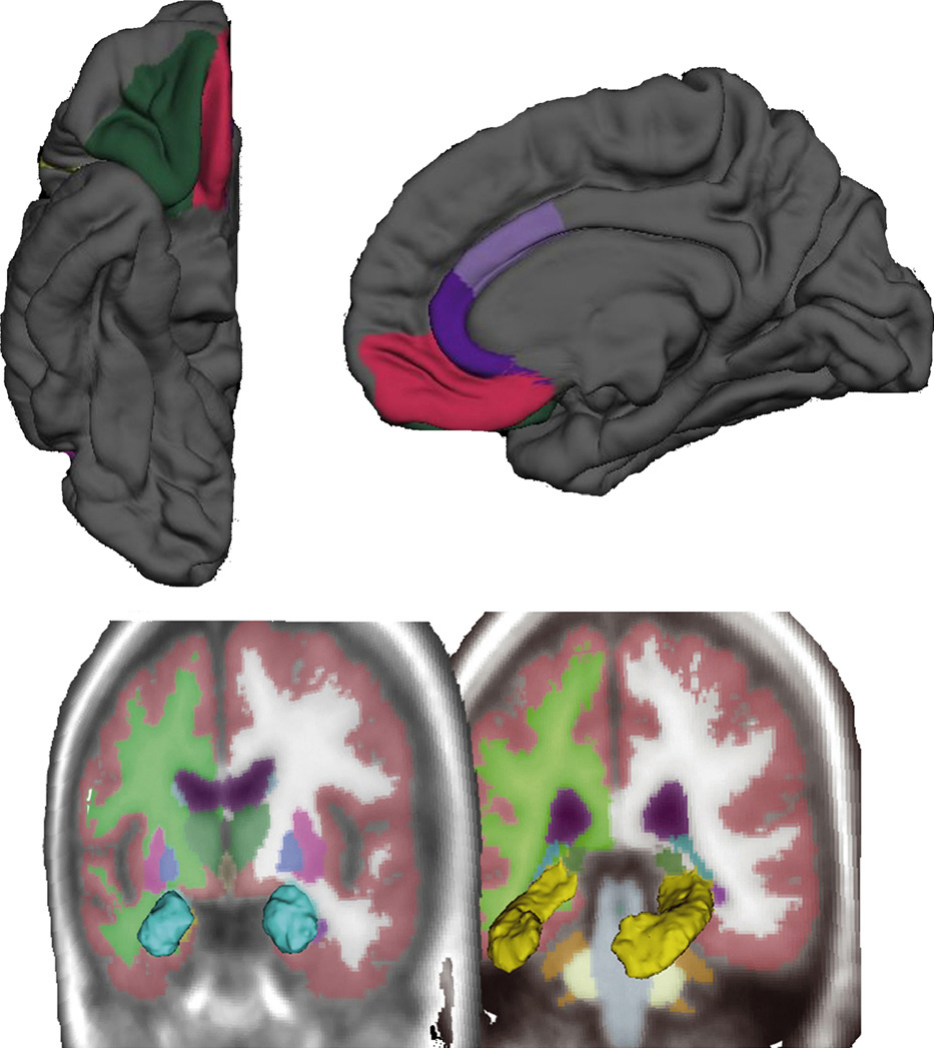
Cortical parcellation units (PUs) involved with a priori hypothesis as obtained by Freesurfer's segmentation. The orbitofrontal cortex was composed by the medial and lateral part, while the anterior cingulate cortex included the subregions rostral- and caudal-anterior cortices. Cortical thickness measurements were extracted from these regions. Neuroimaging analysis was also conducted using volumetric measures of the amygdala and hippocampus (lower panel). Only one hemisphere is shown.

Given the substantial evidence on the neuroanatomical basis of anxiety-like behaviors, the primary aim of this study was to focus multivariate statistical analyses within six regions of interest (ROIs) or parcellation units (PUs) (Cerasa et al. 2010): (1) the orbitofrontal cortex (divided into the medial and lateral parts); (2) the anterior cingulate cortex (divided into rostral- and caudal-anterior cortices); (3) the entire volume of the amygdala and d) the hippocampus. Cortical ROIs or PUs were drawn on maps of average folding patterns on the cortical surface, with reference to an anatomical atlas; while subcortical volumes were extracted by using an automated labeling procedure within Freesurfer (Fischl et al. 2002, 2004) (Fig. 1). Each cortical ROI was mapped back onto each individual subject's unfolded surface by applying the same algorithm that morphed each subject's unfolded surface to the average spherical surface representation in reverse. Mean thickness for each cortical ROI was calculated by averaging the mean cortical thickness measurements at each vertex within a given ROI. Normalized amygdala and hippocampal values were calculated as follows: [raw data volume/ICV]*1000. For each of these ROIs the right- and left-hemisphere measurements were pooled together.

### Voxel-based morphometry

To corroborate Freesurfer-related findings we further performed a voxel-based analysis investigating GM volume changes. Data were processed using the SPM8 software (http://www.fil.ion.ucl.ac.uk/spm), where we applied VBM implemented in the VBM8 toolbox (http://dbm.neuro.uni-jena.de/vbm.html) and incorporated the DARTEL toolbox that was used to obtain a high-dimensional normalization protocol. Images were bias-corrected, tissue classified, and registered using linear (12-parameter affine) and nonlinear transformations, within a unified model. Subsequently, the warped GM segments were affine transformed into MNI space and were scaled by the Jacobian determinants of the deformations (modulation). Finally, the modulated volumes were smoothed with a Gaussian kernel of 10-mm full width at half maximum (FWHM).

### Statistical analysis

The difference in sex distribution was evaluated with chi-square test. The differences between demographical data were assessed using unpaired *t*-test. Correlation analysis was performed using *r*'s Spearman. All statistical analyses had a two-tailed alpha level of <0.05 for defining significance.

### Freesurfer statistical analysis

Association between brain anatomy, obtained by automated method (Freesurfer), and psychological measures were assessed by multiple regression analyses, entering all variables simultaneously. Multiple regression analyses were conducted with automated brain measurements (entire volume of the hippocampus and amygdala, average cortical thickness of the rostral/caudal ACC and medial/lateral OFC) as predictors and anxiety tests as predicted variables. Moreover, age and gender were forced into the model as predictors variables, since previous studies demonstrated correlations between aging and anxiety level (Schneider and Heuft 2012) as well as gender-dependent effects on the relationship between brain anatomy and anxiety (Montag et al. 2012). For this reason, these two demographical variables were used to further test the discriminant validity of association with anxiety. Of note, we also conducted curvilinear regression analysis to verify if volumetric changes may have occurred as a U-shaped course. The multiple regression model, thus, included two separate regressors for each subject: (1) the behavioral measures of anxiety (testing for linear effects) and (2) the square of these values (testing for quadratic functions). Curvilinear regression analysis was reported in the Supplementary Materials (Table S1), showing a very similar pattern of findings as reported for linear regression analysis. The analyses were applied to the whole sample. Anatomical quantitative measurements of ROIs and psychological data were fed into separate regression analyses performed with STATISTICA version 6.0 (http://www.statsoft.com).

### VBM statistical analysis

The GM volume maps were statistically analyzed using the general linear model based on Gaussian random field theory. To assess the relationships between GM volume and anxiety scores, the smoothed and modulated GM images were entered into multiple regression model, with age and gender as covariates of no-interest. As previously stated, we decided to use the amygdala, hippocampus, ACC, lateral and medial OFC as bilateral a priori ROIs given their consolidated role in anxiety disorders (Blackmon et al. 2011; Kuhn et al. 2011; Spampinato et al. 2009; Strawn et al. 2013; Greenberg et al. 2013). All ROIs were created with the “aal.02” atlas included in the Wake Forest University Pickatlas software version 1.04 (http://www.fmri.wfubmc.edu/download.htm). All analyses were thresholded by using correction for multiple comparisons (family-wise error (FWE) *P* < 0.05) within ROIs. Moreover, for exploratory purpose, we also reported findings outside ROIs, considering voxels surviving an uncorrected whole-brain threshold of *P* < 0.001.

## Results

### Demographical data

Demographic features of the healthy individuals are summarized in Table 1. On the HARS, the mean score for the 121 participants was 5.13 (SD 4.52; range 0–21). On the STAI-trait and STAI-state, the average scores for the sample were, respectively, 34.1 (SD 7.28; range 22–56) and 35.73 (SD 8.1; range 19–67). Since it has been demonstrated that women tend to be more anxious than men, we evaluated the influence of this variable in our population. Indeed, women were characterized by higher levels of anxiety (HARS: 5.64 ± 4.48, STAI-state: 35.9 ± 7.6, STAI-trait: 36.8 ± 8.52) than men (HARS: 4.47 ± 4.08, STAI-state: 32.1 ± 5.1, STAI-trait: 33.8 ± 5.8), without reaching a significant threshold (see Table 1).

**Table 1:** Demographic characteristics

	Whole group		
	
Variables	Mean ± SD	Median (range)	*P-*level (*t/U*)
*N*	121		
Women, *n* (%)	67 (54)		
Age (years)	38.7 ± 15.1	36 (21–70)	
Educational level (years)	14.7 ± 3.2	15 (5–21)	
MMSE	29.4 ± 0.9	30 (26–30)	
HARS	5.13 ± 4.52	4 (0–21)	
STAI-state	34.1 ± 7.28	33 (22–56)	
STAI-trait	35.73 ± 8.1	35 (19–67)	

	Gender differences		
	
Variables	Men	Women	*P-*level
*N*	54	67	
Age (years)	39.2 ± 14.4	38.3 ± 15.7	0.76
Educational level (years)	14.4 ± 3.2	14.4 ± 3.6	0.86
MMSE	29.48 ± 0.8	29.4 ± 1.1	0.75
HARS	4.47 ± 4.08	5.64 ± 4.48	0.21
STAI-state	32.1 ± 5.1	35.9 ± 7.6	0.1
STAI-trait	33.8 ± 5.8	36.8 ± 8.52	0.09

Data are given as mean values (SD) and analyzed using Unpaired *t*-test.

MMSE, Mini Mental State Examination; STAI, State-Trait Anxiety Inventory (STAI); HARS: Hamilton Anxiety Rating Scale.

Since previous studies demonstrated a strong association between HARS and STAI scales, we tried to confirm this evidence. Simple regression analysis confirmed that there was a moderate (but significant) correlation between HARS and STAI-state (*r* = 0.24; *P* = 0.006) and very strong correlations between HARS and STAI-trait (*r* = 0.42; *P* < 0.001) and between STAI-trait and STAI-state (*r* = 0.63; *P* < 0.0001). Moreover, since anxiety has been reported to be higher in the elderly (Schneider and Heuft 2012), we tried to confirm this evidence in our large cohort. Simple regression analysis revealed a positive significant association of age with HARS scores (*r* = 0.22; *P* < 0.01) but not with STAI-state (*r* = −0.03; *P* = 0.71) or STAI-trait (*r* = 0.01; *P* = 0.87).

### Freesurfer data

Multiple regression analysis results are presented in Table 2. Anxiety scores, as assessed by HARS, were significantly predicted by anatomical variance of the caudal (*β* = 0.31; *P*-level = 0.01) and rostral ACC (*β* = 0.38; *P*-level = 0.002), where individuals with high HARS scores showed a pronounced cortical thickening. Otherwise, STAI scales were not significantly influenced by any brain region involved in emotional processes. However, multiple regression analysis demonstrated that the best predictor of either STAI-state or STAI-trait scores was gender (*β* = 0.25; *P*-level = 0.01; *β* = 0.23; *P*-level = 0.02).

**Table 2:** Multiple regression analyses contrasting association patterns between theoretically relevant neuroanatomical structures underlying anxiety-like behaviours and individual variables for the whole sample

Predictors		STAI-state	STAI-trait	HARS
Gender	*β*	**0.25**	**0.23**	0.11
	*P*	**0.01**	**0.02**	0.22
Age	*β*	−0.01	0.02	0.18
	*P*	0.91	0.77	0.08
HP	*β*	−0.02	−0.07	−0.06
	*P*	0.78	0.52	0.53
Amygdala	*β*	0.1	0.06	0.03
	*P*	0.34	0.54	0.74
CaudalAnterior-ACC	*β*	0.15	0.15	**0.31**
	*P*	0.21	0.21	**0.01**
RostralAnterior-ACC	*β*	0.19	0.1	**0.38**
	*P*	0.12	0.42	**0.002**
Lateral OFC	*β*	0.12	−0.03	−0.13
	*P*	0.35	0.81	0.31
Medial OFC	*β*	−0.07	−0.07	0.12
	*P*	0.63	0.64	0.39
Adjusted *R*^2^		0.06	0.02	**0.13**

Significant associations are shown in bold.

HP, Hippocampus; ACC, Anterior Cingulate Cortex; OFC: Orbitofrontal Cortex.

### VBM data

**Figure 2:**
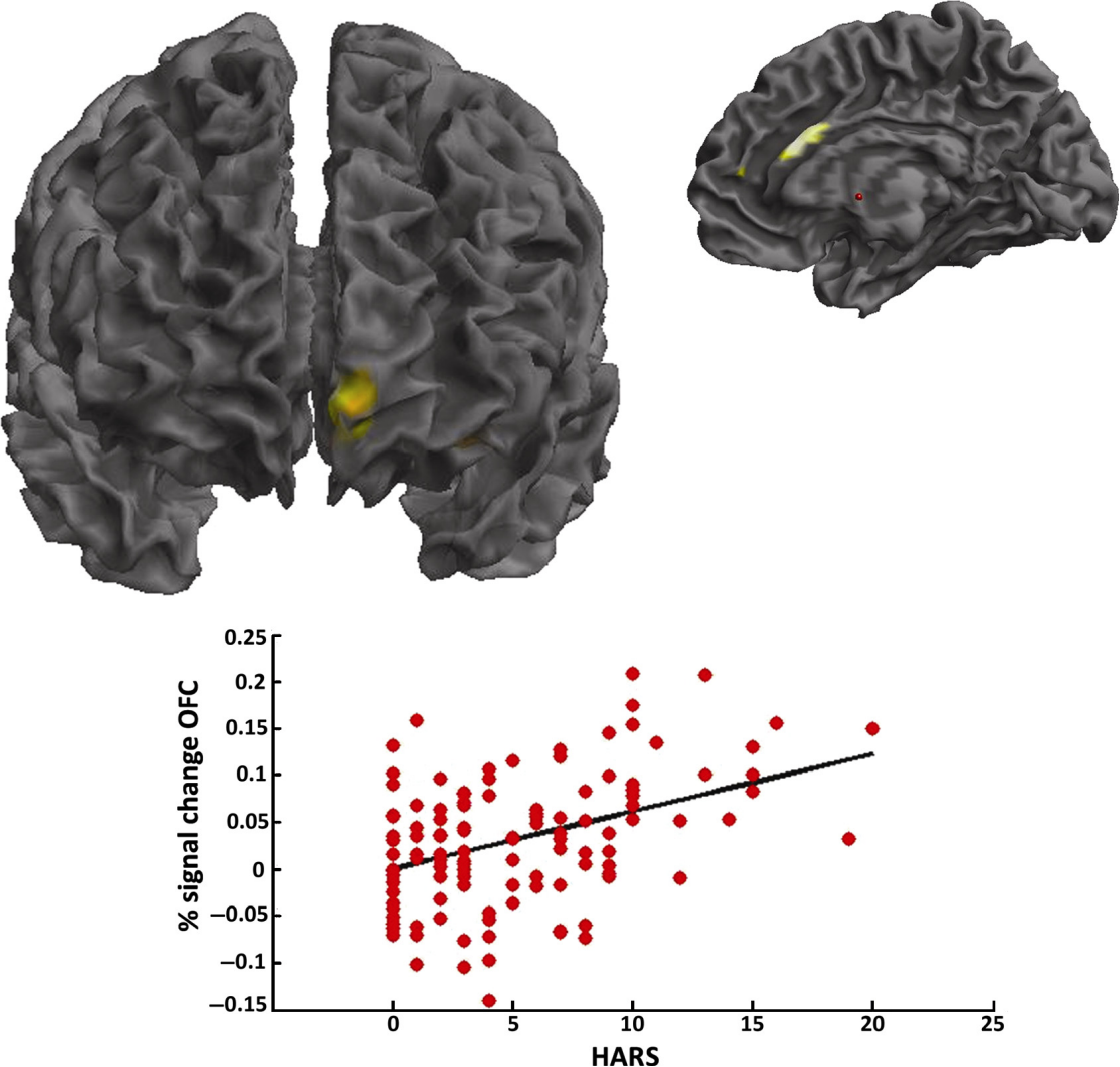
Relationship between the medial OFC and ACC volumes with HARS scores as detected by Voxel-based morphometry (VBM) analysis. Scatterplot with linear fit (solid black line) is also showed in the lower panel (surviving correction for multiple comparisons, FWE < 0.05).

The relationship between anxiety and brain anatomy was also investigated by using simple regression analysis using a voxel-based approach. Voxel-based morphometry (VBM) confirmed the association between HARS measures and GM volume of the ACC (MNI local maxima: *x* = 0, *y* = 48, *z* = 6, *T*-value = 3.61, P_FWE-ROI_ = 0.04), also revealing an additional correlation with the medial OFC (MNI local maxima: *x* = −6, *y* = 58, *z* = −5, *T*-value = 4.2, P_FWE-ROI_ = 0.01) (Fig. 2).

**Figure 3:**
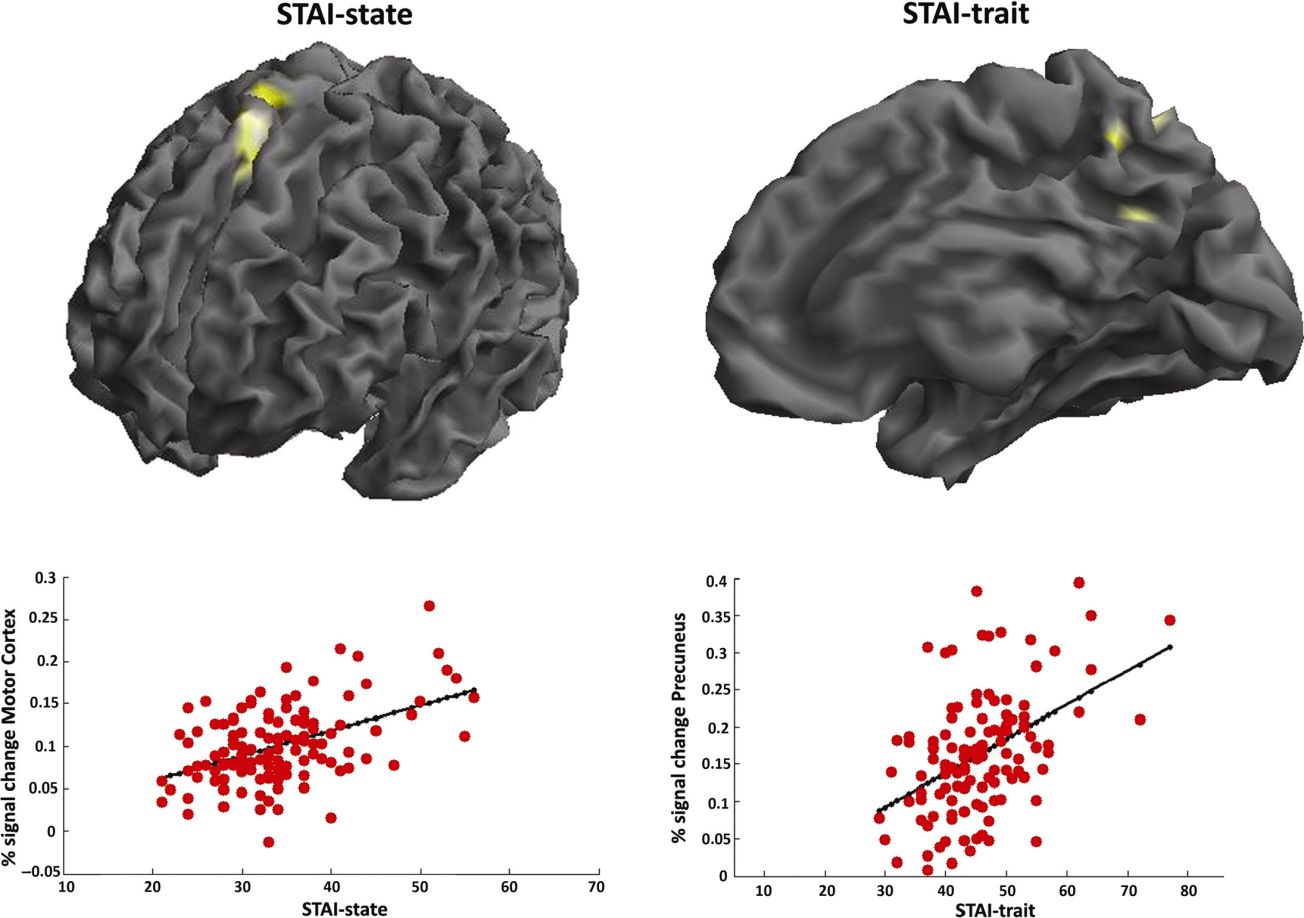
Voxel-based morphometry (VBM) analysis reveals positive correlations (considering an exploratory uncorrected whole -brain statistical threshold of *P* < 0.001) between gray matter volume of the medial premotor cortex and STAI-state scores (left side), as well as between gray matter volume of the precuneus and STAI-trait scores (right side). Scatterplots with linear fit (solid black line) is also showed in the lower panel.

Considering the STAI scales, no significant relationships between anxiety scores and GM volumetric changes were detected within a priori ROIs. Otherwise, outside ROIs, increases in the STAI-state scores corresponded to increased GM volume in a larger cluster encompassing medial motor and premotor cortices (MNI local maxima: *x* = −10, *y* = −18, *z* = 60, *T*-value = 4.36, P_uncorrected_ < 0.001), while STAI-trait scores strongly correlated with GM volume signal changes in the precuneus (MNI local maxima: *x* = −6, *y* = −57, *z* = 12, *T*-value = 4.79, P_uncorrected_ < 0.001) (Fig. 3).

## Discussion

This study is aimed at providing biological correlates of STAI and HARS. Since these scales are widely employed for assessing anxiety in the psychological and clinical realms, one unsolved question is whether these scales embrace a similar psychopathological spectrum or whether these reflect different aspects of the anxiety phenomenon. Our multimodal neuroimaging study would seem to confirm the former hypothesis. In fact, despite a significant intercorrelation among scales scores, the ACC resulted in being the only brain region significantly associated to HARS scores. Otherwise, the STAI scales did not show any significant relationship with anatomical regions underlying anxiety-like behaviors, but their scores were predicted by gender.

Overall, we obtained several pieces of evidence. First, HARS scores were linked to neuroanatomical changes in a brain region strongly involved in emotional regulation: the ACC. Specifically, we found a positive correlation with the entire ACC. It has been proposed that this region plays a critical key role in emotional processing acting like a generator of physiological or behavioral responses (Etkin et al. 2011), and, consequently, this is extensively involved in anxiety-like behaviors and anxiety disorders. In particular, functional studies investigating the neural correlates of anxiety symptoms in healthy individuals demonstrated abnormal activation of the ACC in subclinical anxious subjects during the experience of pain (Ochsner et al. 2006) and during the extinction of conditioned fear (Sehlmeyer et al. 2011). Similarly, structural neuroimaging studies investigating the anatomical predisposition to anxiety traits, reported significant correlation between high-level anxiety (Spampinato et al. 2009) and volumetric variability in the ACC. Other evidence coming from imaging genetic studies in healthy subjects provides additional confirmation for our data, demonstrating a specific link between genetic variations in the serotonin transporter promoter polymorphism (5-HTTLPR) (a neurotransmitter critically involved in emotional regulation) and neural activity of the ACC, demonstrating a genetic liability for anxiety-like behaviors (Drabant et al. 2012). All this evidence suggests that neurobiological alterations underlying ACC have an association with the development of anxious personality traits and may enhance the vulnerability for developing anxiety disorders. Indeed, similar to the evidence described in a healthy population, neuroimaging studies investigating patients with a pathological level of anxiety (GAD and SAD) confirmed the presence of brain abnormalities in the ACC. In particular, Greenberg et al. (2013) showed an abnormal functional response of the ACC in patients with GAD during fear generalization tasks. Schienle et al. (2011) described a positive relationship between GM volume of this area and the self-reporting on the degree of worrying in a GAD group. Another study found an inverse correlation between thickness of the ACC and the severity of the social anxiety symptoms in patients with SAD (Frick et al. 2013). For this reason, given the reported neural abnormalities of the ACC in physiological and pathological conditions, it is possible to define this region as a reliable biomarker that may include some aspects of the psychopathological spectrum of anxiety, and, consequently, HARS scores may represent a reliable behavioral indicator for describing this spectrum. Although multivariate statistical analysis highlighted the prominent relationship between ACC and anxiety symptoms as assessed by HARS, univariate regression analysis performed with VBM depicted the involvement of an additional brain region, the OFC, strongly implicated in anxiety expression (Milad and Rauch 2007). The significant association between thickening of the OFC and increased anxiety in healthy individuals is not surprising since previous works highlighted the key role of this region in the functional integration of emotional information (Blackmon et al. 2011).

Then, despite a correlation between the two scales, the multivariate and multimodal neuroimaging analyses revealed that the anatomical variability in the limbic networks did not contribute to the STAI measurements. The lack of significant findings might dependent upon the fact that STAI scores do not strictly evaluate anxiety per se (Kennedy et al. 2001; Bados et al. 2010), but rather, negative affects and personality traits capturing several dimensions of emotional negativity, including anxiety (Bieling et al. 1998). Personality traits are individual characteristics that influence cognition, emotions, and behavior. The relationship between personality traits with anxiety is complex. Different personality tests embrace a variety of personality domains trying to describe aspects of an individual's character that influence cognition, emotions, and behavior, leading to adaptive or maladaptive responses. For instance, neuroticism, one of the most consistent traits of the human personality, assessed by NEO-Personality Inventory on the basis of the Five Factor Model of personality, captures several dimensions of emotional negativity, including anxiety (Costa and McCrae 1992). Indeed, a correlation between the STAI scale and the measure of neuroticism has been demonstrated (Gonda et al. 2009). Similarly, harm avoidance, a personality trait of temperament and character inventory (TCI) (Cloninger et al. 1993) referred to an individual's tendency to inhibit behaviors, apprehensiveness and pessimism, has been found to be positively correlated with anxiety and depression status (Minelli et al. 2009; Uliaszek et al. 2009). Investigating functional basis of personality traits, Adelstein et al. (2011) found a significant connectivity between the prefrontal cortex and the precuneus, modulated by the neuroticism level. Again, some neuroimaging studies showed that harm avoidance relies on other specific brain structures linked to personality traits, including the precuneus, cerebellum, and basal ganglia (Laricchiuta et al. 2013a, 2013b; Gardini et al. 2009). For this reason, it could be hypothesized that neural correlates of STAI scores rely on more widespread neural networks, outside limbic circuitries. Our additional neuroimaging data would seem to confirm this hypothesis. In fact, considering regression analysis performed with VBM, we detected an association between STAI-trait measures and GM volume of the precuneus. Unfortunately, this finding did not survive correction for multiple comparisons, thus making speculative any attempt to delineate definitive conclusions on the relationship between anxiety trait, as assessed by STAI, and the role played by the precuneus (self-consciousness, Fuentes et al. 2012). However, it could bear in mind that several previous neuroimaging studies described a significant relationship between the anatomy of the amygdala, OFC, and ACC and increasing values of STAI scores (Blackmon et al. 2011; Baur et al. 2012; Kuhn et al. 2011; Spampinato et al. 2009). The discrepancy between our data and the present literature may be due to some methodological differences. Apart from the different statistical model (multiple regression model, including age and gender as predictors of anxiety level together with thickness measurements of specific brain regions), in all previous neuroimaging studies, investigating the neural correlates of anxiety, the employed nonclinical samples were very small (Baur et al. (2012), included 32 healthy controls; Spampinato et al. (2009), included 30 healthy controls; Blackmon et al. (2011), included 34 healthy controls; Kuhn et al. (2011) included 34 healthy controls). The only neuroimaging study investigating a large cohort (Montag et al. 2012) suggested that the relationship between anxiety and brain anatomy is critically influenced by gender, a critical variable not considered in previous studies cited above. This latter finding perfectly agrees with our data demonstrating that STAI scores are more influenced by gender rather than by anatomical variability in the limbic regions (Table 2).

Generally, STAI and HARS measurements did not differ only for neural correlates, but are fundamentally characterized by important psychometric differences. As previously argued by Mondolo et al. (2007) the specific instructions of the two questionnaires differ. On the HARS, there is a description of somatic and psychological feelings. The clinician rates the patient by finding the answer that best describes the extent to which he/she has these conditions. In contrast, on the STAI-state the patient is instructed to self-complete the scale in order to indicate how he/she feels right at that moment, while on the STAI-trait the patient is instructed to indicate how he/she generally feels. A second factor is the number of items in the two questionnaires. The HARS consists of only 14 questions, compared to the 40 questions in the STAI. This difference is related to the fact that the HARS is an efficient screening instrument for assessing clinically significant degrees of anxiety. In contrast, the STAI-trait scale represents a personality inventory, because it evaluates the existence of stable individual differences in the tendency to respond with state anxiety in the anticipation of threatening situations. In this sense, the STAI-trait scale is administered to evaluate psychopathological anxiety traits and is not a screening test for anxious mood disorders (Mondolo et al. 2007). For this reason, it is possible to hypothesize that the STAI-related assessment of anxiety is more dependent upon participant's ability to comment or report on his or her own mental state, than those performed by HARS. This well-known cognitive function is called metacognition, that drastically might influence interpretation of personality inventories (Kanai and Rees 2011). Future studies investigating the impact of metacognitive functions in modulating assessment of anxiety traits are warranted to demonstrate this hypothesis. Similarly, further investigations considering other scales, such as the BIS/BAS (Carver and White 1994), which has been demonstrated to be highly reliable and valid scale of anxiety (Zinbarg and Mohlman 1998), are warranted.

## Conclusion

To our knowledge, this is the first study that provides the neural correlates of two important behavioral measures for assessing anxiety in a large nonclinical population using a multivariate statistical approach. The HARS scale, mainly used in the clinical approach, is related to brain structure strongly involved in anxiety disorders and anxiety-like behaviors, such as the ACC, while the STAI scales, widely used in both psychological and clinical contexts, does not show evident relationships with limbic regions, thus suggesting that this scale is more closely linked to certain personality traits (i.e., harm avoidance or neuroticism) in which anxiety is considered as a sub-dimension. We retain that our findings might provide a useful landmark for helping clinical practice.

## Supporting Information

**Table S1.** Multiple regression analyses contrasting nonlinear association patterns between theoretically relevant neuroanatomical structures underlying anxiety-like behaviors and individual variables for the whole sample.
