# Supplementary material for: The neuroanatomical correlates of anxiety in a healthy population: differences between the State-Trait Anxiety Inventory and the Hamilton Anxiety Rating Scale
Source: Brain Behav. 2014 Jun 18;4(4):504–14. doi: 10.1002/brb3.232 (PMC4128032; doi:10.1002/brb3.232)
Supplement: Supplementary file 1 — Table S1. Multiple regression analyses contrasting nonlinear association patterns between theoretically relevant neuroanatomical structures underlying anxiety-like behaviors and individual variables for the whole sample. [file brb30004-0504-SD1.doc]

**ONLINE SUPPLEMENTARY MATERIAL**

**The neuroanatomical correlates of anxiety in healthy population: differences between the State-Trait Anxiety Inventory and the Hamilton Anxiety Rating Scale**

Giulia Donzuso^1,3*^, Antonio Cerasa^1*§^, Maria Cecilia Gioia^1^, Manuela Caracciolo^1^, Aldo Quattrone^1,2^

^1^ Neuroimaging Research Unit, Institute of Neurological Sciences, National Research Council, Germaneto (CZ), Italy

^2^ Institute of Neurology, University “Magna Graecia”, Germaneto (CZ), Italy

^3^ Department “G.F. Ingrassia”, Section of Neuroscience, University of Catania, Catania, Italy

^§^ Address correspondence to: **Antonio Cerasa**, PhD

Neuroimaging Research Unit,

Institute of Neurological Sciences, National Research Council, Germaneto (CZ), Italy

Tel: +39-0961-3695904; Fax: +39-0961-3695919; e-mail: [a.cerasa@unicz.it](mailto:a.cerasa@unicz.it)

**Table S1: Multiple regression analyses contrasting non linear association patterns between theoretically relevant neuroanatomical structures underlying anxiety-like behaviours and individual variables for the whole sample**

| **Predictors** |  | **STAI-state** | **STAI-trait** | **HARS** |
| --- | --- | --- | --- | --- |
| **Sex** | Beta | **0.24** | **0.25** | 0.13 |
|  | *P* | ***0.01*** | ***0.01*** | *0.17* |
| **Age** | Beta | 0.04 | 0.03 | 0.2 |
|  | *P* | *0.68* | *0.75* | *0.06* |
| **HP** | Beta | -0.01 | -0.04 | 0.01 |
|  | *P* | *0.9* | *0.81* | *0.92* |
| **Amygdala** | Beta | 0.05 | 0.04 | -0.01 |
|  | *P* | *0.58* | *0.68* | *0.96* |
| **CaudalAnterior-ACC** | Beta | 0.24 | 0.26 | **0.51** |
|  | *P* | *0.2* | *0.17* | ***0.007*** |
| **RostralAnterior-ACC** | Beta | 0.36 | 0.14 | **0.59** |
|  | *P* | *0.1* | *0.5* | ***0.006*** |
| **Lateral-OFC** | Beta | 0.3 | 0.06 | -0.14 |
|  | *P* | *0.29* | *0.83* | *0.61* |
| **Medial-OFC** | Beta | -0.17 | -0.15 | 0.27 |
|  | *P* | *0.58* | *0.64* | *0.38* |
| **Adjusted R^2^** |  | 0.05 | 0.02 | **0.14** |

Significant associations surviving corrections for multiple comparisons are shown in bold.

HP: Hippocampus

ACC: Anterior Cingulate Cortex

OFC: Orbitofrontal Cortex
